# Prokaryotic communities associated with marine hydrothermal systems of the Gulf of California

**DOI:** 10.3389/fmicb.2024.1501893

**Published:** 2025-01-17

**Authors:** Ruth Noemí Aguila-Ramírez, Bárbara González-Acosta, Karla María Gutiérrez-Almada, José Manuel Borges-Souza, Rocío Guadalupe Cervantes-Gámez, Eduardo Quiróz-Guzmán

**Affiliations:** ^1^Instituto Politécnico Nacional-Centro Interdisciplinario de Ciencias Marinas, La Paz, Mexico; ^2^Departamento de Estudios para el Desarrollo Sustentable de Zonas Costeras, Centro Universitario de la Costa Sur de la Universidad de Guadalajara, Departamento de Estudios para el Desarrollo Sustentable de Zonas Costeras, Autlán, Mexico; ^3^Centro de Investigaciones Biológicas del Noroeste, La Paz, Mexico

**Keywords:** shallow-water hydrothermal vent, sediment, diversity, bacterial diversity, prokaryotic community

## Abstract

**Introduction:**

Marine hydrothermal systems (MHS) are considered extreme environments due to their unique physicochemical conditions, which are challenging for most organisms. This study investigates the microbial communities in three MHS sites in Baja California Sur, Mexico.

**Methods:**

Sediment samples were collected in two seasons of the year: rainy and dry season. Bacterial DNA was extracted, the V3-V4 regions of the 16S rRNA gene were amplified.

**Results and discussion:**

The analysis of microbial community structure and composition revealed that species richness and diversity were higher at control sites (not influenced by hydrothermal conditions). Samples from the MHS showed temporal variation in richness, as measured by the Chao1 index. *Alphaproteobacteria* and *Gammaproteobacteria* were the dominant classes. No significant differences in community structure were found between the seasons or between the control and MHS sites. However, the analysis did reveal differences in community structure among the three hydrothermal locations: Burro, Santispac, and Agua Caliente. The presence of *Gammaproteobacteria*, *Alphaproteobacteria*, *Deltaproteobacteria*, and *Betaproteobacteria* highlights their key roles in primary production within shallow hydrothermal systems, these microbial communities demonstrate their capacity to colonize diverse substrates. This study enhances the microbiological understanding of hydrothermal environments in Baja California Sur, and molecular analysis of unculturable microbes could provide further insights into their physiology and ecological roles in shallow hydrothermal systems.

## 1 Introduction

Marine hydrothermal systems (MHS) are extreme environments because the fluids emitted from vents or fissures in the marine sediment present unusual physical–chemical conditions, such as high temperatures, high concentrations of dioxide carbon, hydrogen sulfide, hydrocarbons, heavy metals, and other unfavorable conditions for most eukaryotic organisms (Lentini et al., 2014). MHS is associated with seafloor spreading zones, commonly occurring where two tectonic plates move (Ramasamy et al., 2020). While shallow hydrothermal systems (SHT) share similar internal structures and fluid characteristics with deep-sea vents, they differ in several ways, including biomass, temperature, pH levels, and other factors. Fluids from SHT may be more influenced by sunlight and interaction with surface waters, affecting their chemical composition. Sulfur and other compounds may be lower due to dilution by surrounding water. The limited depth of SHT allows their plumes to extend to the nearby area and the water’s surface, facilitating significant interactions with human activities and surrounding ecosystems. SHT in coastal regions are ideal for hydrothermal research because of their strong connection to human activities. These aspects highlight the importance of studying SHT (Zhang et al., 2020). In these environments, few groups of organisms predominated and can sometimes survive due to symbiotic associations (Grzymski et al., 2008). In Baja California Sur (BCS), Mexico, areas of shallow hydrothermal systems have been recorded, the presence of hydrothermal manifestations on the coasts of Bahía Concepción BCS is associated with the extensive tectonic regime that generated the formation of the Gulf of California, comprising phenomena of cortical extension and volcanism, which formed a system of faults on the east coast of the Baja Peninsula. California. The Fault called El Requesón, originates shallow hydrothermal vents and the geothermal springs, running in an NW-SE direction, within the Concepción Peninsula and passing through the coasts of Mapachitos within the Bay (McFall, 1968). According to Prol-Ledesma et al. (2004), the fluids of the hydrothermal systems in this area are enriched with various elements and dissolved compounds, such as Ca, Mn, Si, Ba, B, As, Hg, I, Fe, Li, HCO_3_ and Sr. and low concentrations of Cl, Na, Br and SO_4_, while the gases emitted are mainly N_2_ (54%) and CO_2_ (44%), with lower amounts of CH_4_ (2.2%), Ar (0.7%), O_2_ (0.2%), He (0.04%), and H_2_ (0.007%) (Forrest et al., 2005). Ventana is located south of the city of La Paz, BCS, it has a large expanse of sandy beaches on the Gulf of California and hydrothermal manifestations have been found in several extensions of its coastline like Agua Caliente beach. It is a very little-studied area for which there are no environmental records.

The taxonomic composition of microbial communities inhabiting SHT has been investigated in several areas, including the Aeolian archipelago, Panarea and Vulcan islands (Maugeri et al., 2002; Gugliandolo et al., 2003; Bortoluzzi et al., 2017), considering different typing of substrates (water column, sediments), and show the presences of different phyla of archaea and bacteria (Hirayama et al., 2007; Rajasabapathy et al., 2014; Gugliandolo and Maugeri, 2019).

The number of studies on this topic has recently increased thanks to advances in molecular techniques. The characterization of microbial communities using these molecular techniques and bioinformatic analyses offers a large amount of information, thus facilitating the study of prokaryotic communities (microbiome) through the analysis of genes such as 16S rRNA in bacteria, from environmental samples, evaluating, in particular, the genomic composition and diversity (within and between) of the microbial communities through sequencing technologies, obtaining a greater understanding of the bacterial diversity of sampled areas, including both the cultivable fraction and the non-cultivable (Segata et al., 2013; Silva et al., 2021).

From an ecological perspective, knowledge of the microbiome of a particular environment could allow us to understand various aspects of the microbial life generated there. Therefore, in this work, a prospective study was carried out to characterize bacterial communities associated with three shallow hydrothermal systems with different characteristics in the Gulf of California to the knowledge of their diversity and richness in these environments, and to compare the microbial composition in marine environments not influenced by these extreme conditions.

## 2 Materials and methods

### 2.1 Study site and sample collection

**Figure 1:**
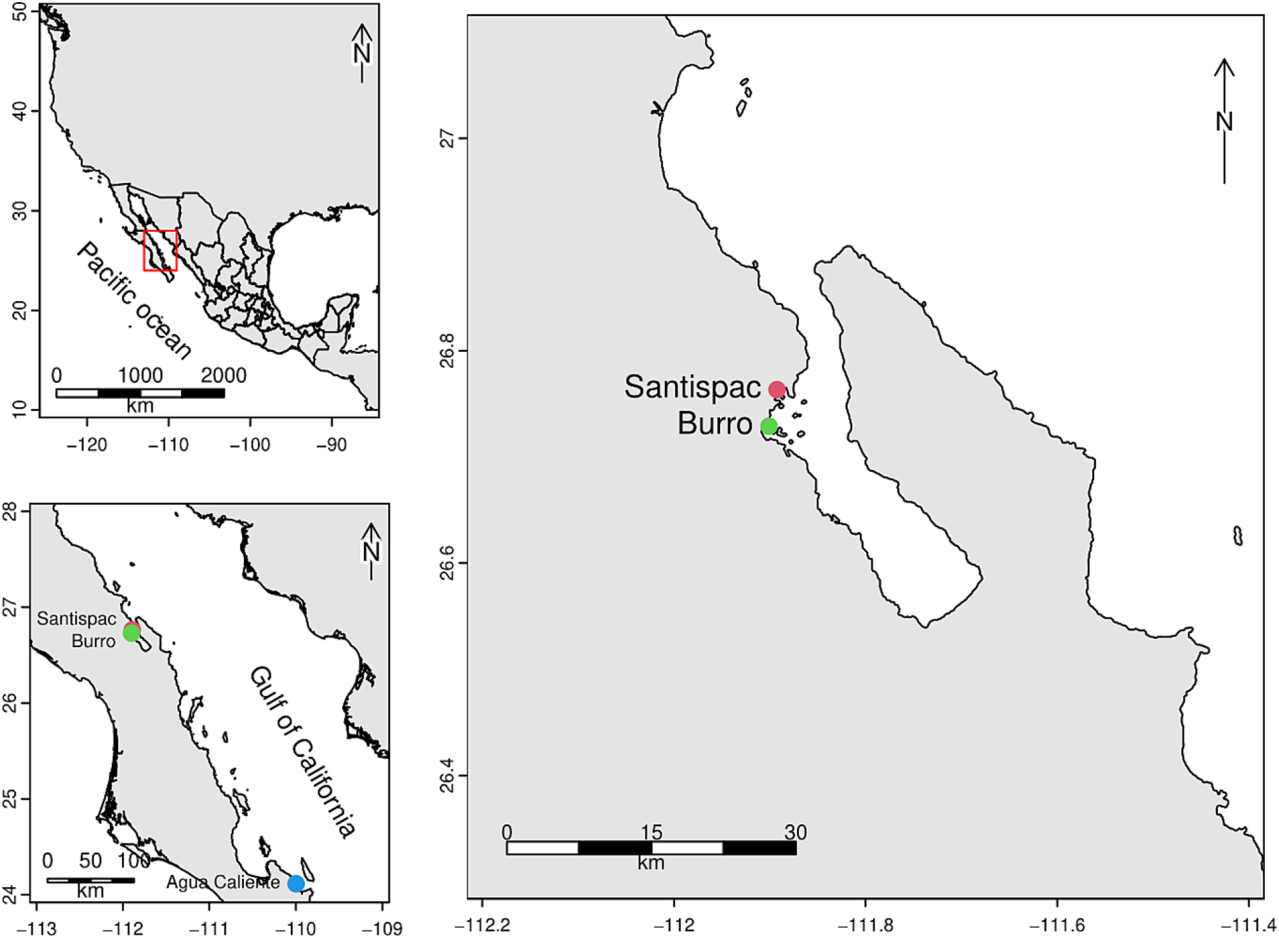
Location of the study areas corresponding to the shallow hydrothermal systems of Baja California Sur.

Three sampling locations with SHT characteristics were delimited in the state of Baja California Sur, Mexico: Burro (B), Bahía Concepción (26° 45′ 48”N, 111° 53′ 33” W), Santispac Mangrove (M), Bahía Concepción (26° 43′ 44”N, 111° 54′ 03” W) and Agua Caliente beach, Ventana (V) (24° 06′ 49”N, 109° 59′ 53” W) (Figure 1).

At each location, the temperature of the sediment was taken using a field thermometer. The sediment was collected in triplicate from the three sites using 15 cm high plastic nucleators, previously washed with chlorine and sterilized with UV light, and was carried out with sterile polyethylene bags. These samples were taken from the cracks observed in the sediment or from where the highest temperatures were recorded (if no cracks were observed in the sediment). In the case of the Burro marine station (B), samples were taken by SCUBA diving at 10–15 m depth. In Santispac mangrove (M), sediment samples were collected at 1–2 m depth. Sampling in Agua Caliente beach (V) was conducted at the lowest tide, approximately two meters from the coastline, digging one and a half meters deep. As control (C) samples, a collection of sediment was made in areas close to the SHT to ensure no influence of the hydrothermal fluids, with the methodology described above, to compare prokaryotic communities from extreme and usual conditions.

The triplicate samples were mixed to obtain a composite sample, and 1 g of sediment from each site was placed in collection tubes to preserve the sample (DNA/RNA Shield-Fecal Collection Tube from Zymo Research). The samples were refrigerated at 4°C until shipment for sequencing analysis by Zymo Research, California, United States.

### 2.2 DNA extraction and sequence analysis

The DNA extraction and sequencing of the samples were carried out through an external service with the ZymoBIOMICS™ Service-Targeted Metagenomic Sequencing (Zymo Research, CA).

ZymoBIOMICS™ DNA Miniprep Kit was used to extract DNA using an automated platform. DNA quality was assessed with NanoDrop before library preparation. Bacterial 16S ribosomal RNA gene-targeted sequencing was performed using the Quick-16S™ NGS Library Preparation Kit (Zymo Research, Irvine, CA). The bacterial 16S primers amplified the V3–V4 region of the 16S rRNA gene. These primers have been custom-designed by Zymo Research to provide the best coverage of the 16S gene while maintaining high sensitivity. The primer set used in this project was Quick-16S™ Primer Set V3–V4 (Zymo Research, Irvine, CA). The sequencing library was prepared using a library preparation process in which PCR reactions were performed in real-time PCR machines to control cycles and prevent PCR chimera formation. The final PCR products were quantified with qPCR fluorescence readings and pooled together based on equal molarity. The final pooled library was cleaned with Select-a-Size DNA Clean & Concentrator™, then quantified with TapeStation^®^ and Qubit^®^. The ZymoBIOMICS^®^ Microbial Community Standard (Zymo Research, Irvine, CA) was used as a positive control for each DNA extraction if performed. The ZymoBIOMICS^®^ Microbial Community DNA Standard (Zymo Research, Irvine, CA) was used as a positive control for each targeted library preparation. Negative controls (i.e., blank extraction control, blank library preparation control) were included to assess the level of bioburden carried by the wet-lab process. The final library was sequenced on Illumina^®^ MiSeq™ with a v3 reagent kit (600 cycles). The sequencing was performed with a 10% PhiX spike-in.

### 2.3 Bioinformatic analyses

Analysis of microbial community structure and composition was conducted on the Illumina MiSeq platform, whose data were processed using the MOTHUR v. 1.48.0 software package (Schloss et al., 2009). All sequences were quality filtered and aligned using the SILVA v138 database from the Microbial Genomics and Bioinformatics Group at the Max Planck Institute for Marine Microbiology, Germany (Quast et al., 2013; Yilmaz et al., 2014), which were further clustered into amplicon sequence variants (ASVs) at 100% sequence identity. Alpha diversity was calculated using the Shannon diversity index (H′) and the Chao1 richness estimator. Beta diversity was calculated using the Bray–Curtis dissimilarity metric and visualized with principal coordinate analysis (PCoA) plots. Analysis of molecular variance (AMOVA) was used to determine whether the clustering within the ordinations is statistically significant at *p < 0.05*. A heatmap was also generated showing the relative abundance of ASVs assigned at the species level, which were classified using the EzBioCloud database (Yoon et al., 2017). Sequences were deposited in the NCBI Short Read Archive under Bio-Project accession number: PRJNA1023234.

### 2.4 Statistical analysis

Regarding bacterial community composition and structure, the molecular variance (AMOVA) test analysis, implemented in MOTHUR software package (Schloss et al., 2009), was applied to determine whether the clustering within the ordinations is statistically significant.

## 3 Results

### 3.1 Temperature of the MHS

In Burro (B), the temperature recorded varied from 75°C to 85°C. This was taken in the hydrothermal fluid emanating from cracks in the sediment at a depth of 10 to 15 meters.

In Santispac mangrove (M), the hydrothermal fluid temperature recorded was between 50 to 75°C, this temperature increased according to the depth in the sediment. In Agua Caliente beach, Ventana (V) the temperature recorded from the SHT ranged from 45 to 70°C.

### 3.2 Bacterial community composition

After quality trimming and filtering, the library size of each sample was normalized to the smallest number of sequences obtained from all stations (4,498 sequences) to minimize any bias due to the difference in the total number of sequences. Generally, the number of OTUs in samples is around 107 to 2,767 OTUs. Although Shannon diversity index and Chao1 richness estimators demonstrated that samples of CBR, CMR, CVD, MD, and VR had higher bacterial diversity and richness (Table 1).

**Table 1:** Bacterial diversity and richness in samples of Burro, Santispac mangrove, and Agua Caliente, Ventana.

Treatment	Total No. OTUs	Shannon diversity index	Chao richness estimator
BD	2001	6.30 ± 0.07	4555.54 ± 14
BR	623	5.49 ± 0.18	715.64 ± 28
CBD	1,320	5.73 ± 0.16	1877.03 ± 22
CBR	1813	6.95 ± 0.75	2257.74 ± 14.2
MD	820	4.53 ± 0.37	1154.29 ± 19
MR	536	5.32 ± 0.15	635.39 ± 26
CMR	2,767	7.39 ± 0.39	4129.49 ± 12
CMD	1,581	5.69 ± 0.23	3279.51 ± 35
VD	107	3.35 ± 0.13	174.02 ± 22
VR	341	5.03 ± 0.22	489.18 ± 18
CVD	749	5.97 ± 0.19	938.47 ± 30
CVR	175	4.44 ± 0.22	235.30 ± 33

BD, Burro dry season; BR, Burro rainy season; CBD, Burro dry season control; CBR, Burro rainy season control; MD, Santispac dry season; MR, Santispac rainy season; CMR, Santispac dry season control; CMD, Santispac rainy season control; VD, Agua Caliente, Ventana dry season; VR, Agua Caliente, Ventana rainy season; CVD, Ventana dry season control; CVR, Ventana rainy season control.

It was observed that species richness, as well as diversity, were higher in control localities, except for the Burro sample in the dry season (BD) and the Agua Caliente, Ventana sample in the rainy season (VR), which were more diverse than in the control zone (CBD and CVR, respectively). In general, the highest diversity values, considering only the SHT samples, were those obtained from the Burro site in the dry season (BD), while the samples that presented less diversity were from Agua Caliente, Ventana in the dry season (VD) (Table 1).

Diversity in Burro during the dry season (BD) was higher in the SHT compared to its control zone at the same season (CBD), otherwise during the rainy season, since the most significant diversity was found in the control zone (CBR). Concerning the Santispac mangrove site, variations in diversity are observed depending on the season; it was greater in the rainy season for both the SHT and control sites. Samples from control sites presented greater diversity than those from SHT, in the rainy season. Ventana was the site with the greatest differences in diversity values between SHT sites by season and with their respective control; for SHT sites, the dry season (VD) presented less diversity according to the Shannon Diversity Index (3.35), while it was observed higher values for the rainy one (VR; 5.03). Regarding the control, the dry season (CVD) presented greater diversity (5.97) than the rainy season (CVR; 4.44), and this was the only control site with less diversity than the SHT one since in all the sites and seasons, the control sites presented greater diversity than the SHT ones (Table 1).

In general, all samples from the SHT showed a temporal variation in the richness estimated by Chao1. The samples from Burro presented a difference during the two seasons analyzed, the same can be observed for the Santispac mangrove with a higher richness estimate during the dry season (MD). In Agua Caliente, the least richness was found during the dry season (VD) (Table 1).

**Figure 2:**
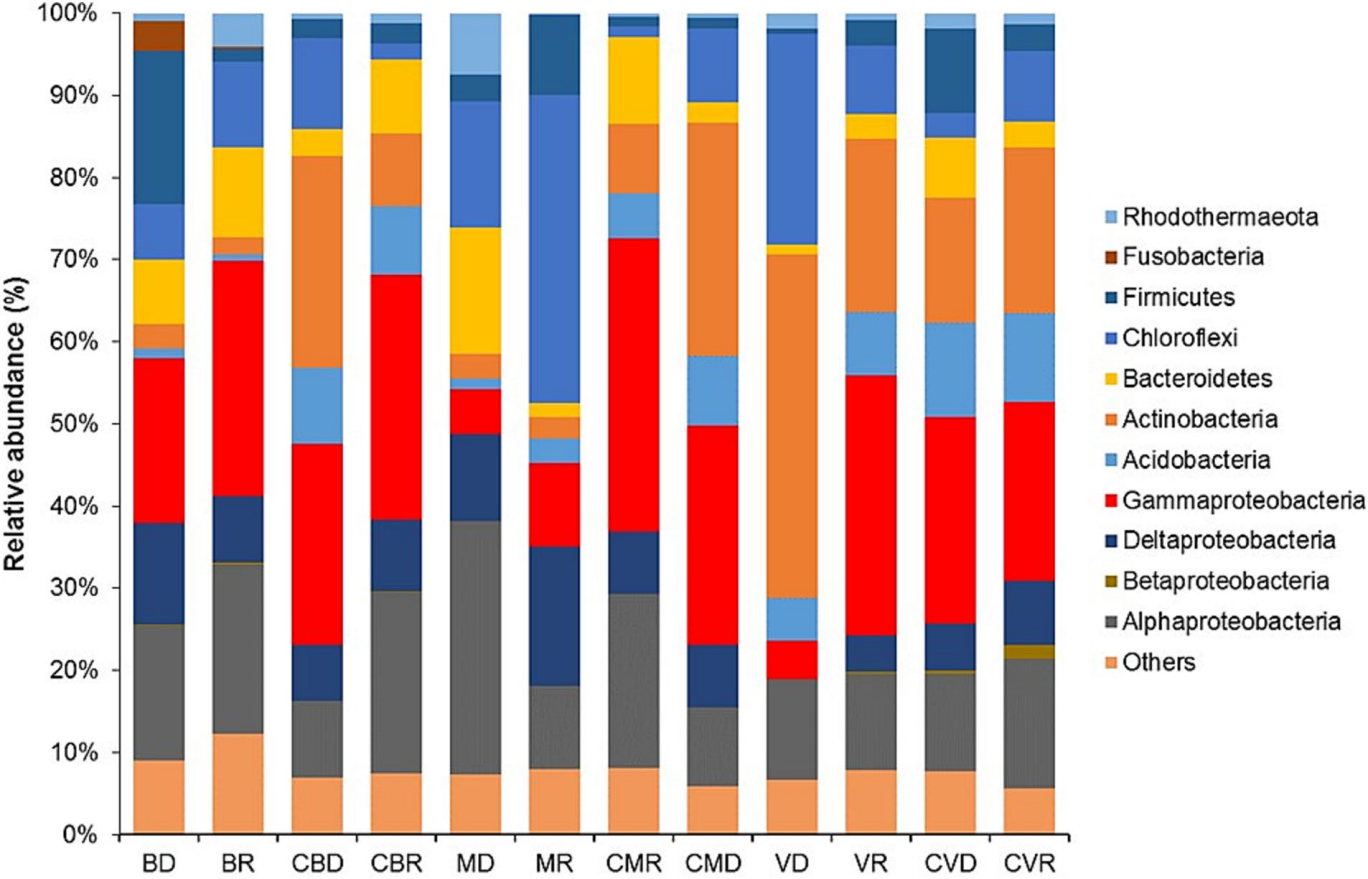
Relative abundance of dominant bacteria linage (phyla; and class for *proteobacteria*) found in BD, Burro dry season; BR, Burro rainy season; CBD, Burro dry season control; CBR, Burro rainy season control; MD, Santispac dry season; MR, Santispac rainy season; CMR, Santispac dry season control; CMD, Santispac rainy season control; VD, Agua Caliente, Ventana dry season; VR, Agua Caliente, Ventana rainy season; CVD, Ventana dry season control; CVR, Ventana rainy season control.

The characterization of the bacterial community was estimated at the phylum level, with *Proteobacteria* being the only group that was classified at the class level (Figure 2). In all the groups, this phylum was found to be the most abundant (*alpha*, *beta*, *delta*, and *gamma proteobacteria*) members of *Gammaproteobacteria* in the station’s BD, BR, CBD, CBR, and *Alphaproteobacteria* in all samples. Members belonging to the phylum *Actinobacteria* were also observed in a high percentage in VD, VR, CVD, and CVR. Although members belonging to the phylum *Firmicutes* were detected in major proportions in BD, MR, and CVD, their abundances were reduced in the rest of the groups (Figure 2). Likewise, a significant increase (*p* < 0.05) of members belonging to the phylum *Chloroflexi*, with a greater abundance in the station MR.

Moreover, members belonging to the phylum *Bacteroidetes* and class *Deltaproteobacteria* increased their relative abundance in all stations (37% ± 1.24 and 28.6% ± 2.01, respectively), regardless of the zone of isolation.

**Figure 3:**
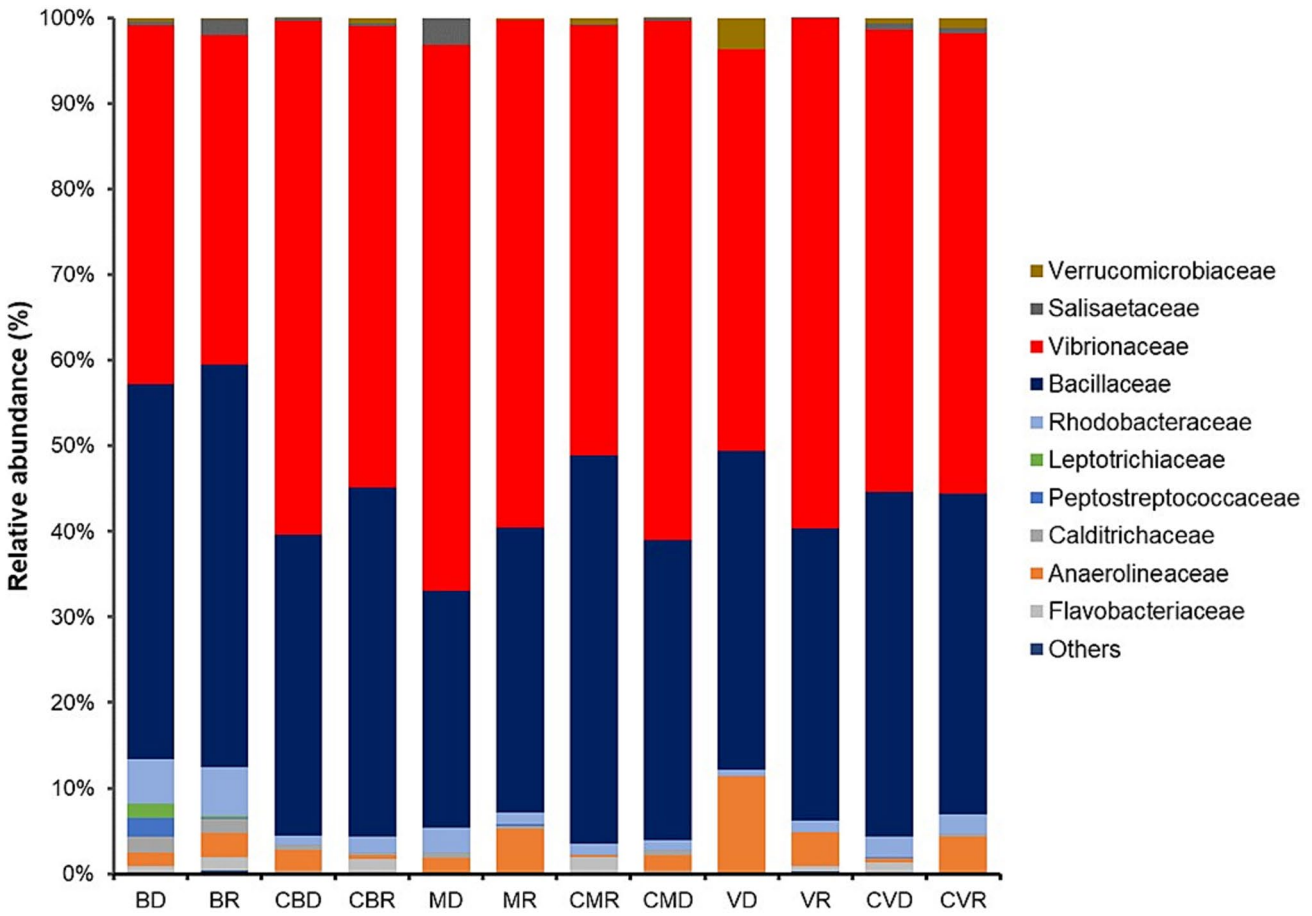
Relative abundance of dominant bacterial families found in BD, Burro dry season; BR, Burro rainy season; CBD, Burro dry season control; CBR, Burro rainy season control; MD, Santispac dry season; MR, Santispac rainy season; CMR, Santispac dry season control; CMD, Santispac rainy season control; VD, Agua Caliente, Ventana dry season; VR, Agua Caliente, Ventana rainy season; CVD, Ventana dry season control; CVR, Ventana rainy season control.

Interestingly, a higher proportion of *Alphaproteobacteria* (particularly members belonging to the family *Vibrionaceae* and *Rhodobacteraceae*) and *Firmicutes* (particularly members belonging to the family Bacillaceae) (Figure 2) was found in all stations. A slight increase in the abundance of *Calditrichaceae* was observed in BD, and BR (Figure 3).

### 3.3 Bacterial community structure

**Figure 4:**
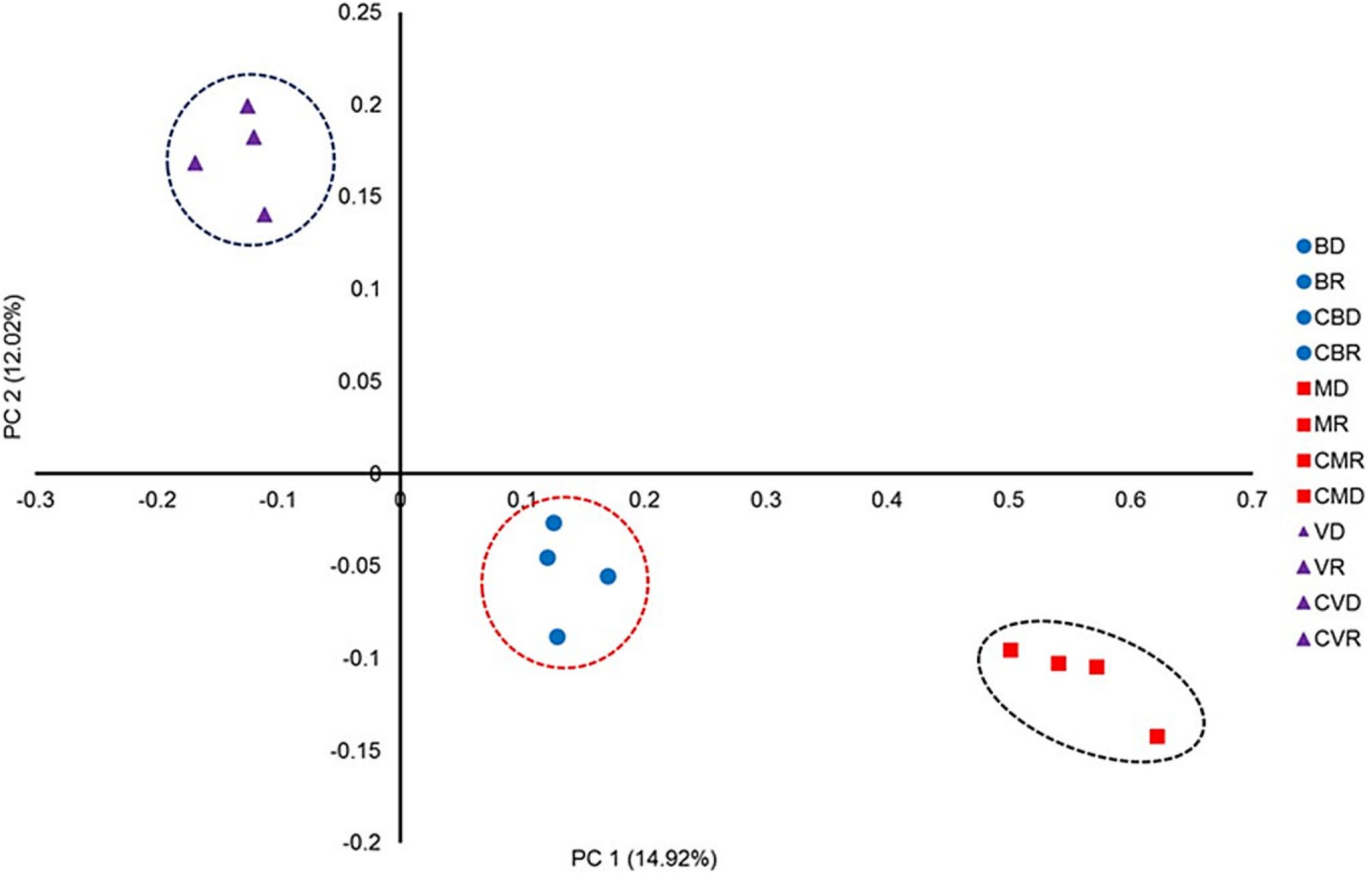
Bacterial community structures of SMHS station samples from BD, Burro dry season; BR, Burro rainy season; CBD, Burro dry season control; CBR, Burro rainy season control; MD, Santispac dry season; MR, Santispac rainy season; CMR, Santispac dry season control; CMD, Santispac rainy season control; VD, Agua Caliente, Ventana dry season; VR, Agua Caliente, Ventana rainy season; CVD, Ventana dry season control; CVR, Ventana rainy season control. PCoA plots are based on the Yue & Clayton measure of dissimilarity (Yue and Clayton, 2005). Ellipses represent the 95% confidence intervals. The first and second axes represent 14.92 and 12.02% of the variation, respectively.

**Figure 5:**
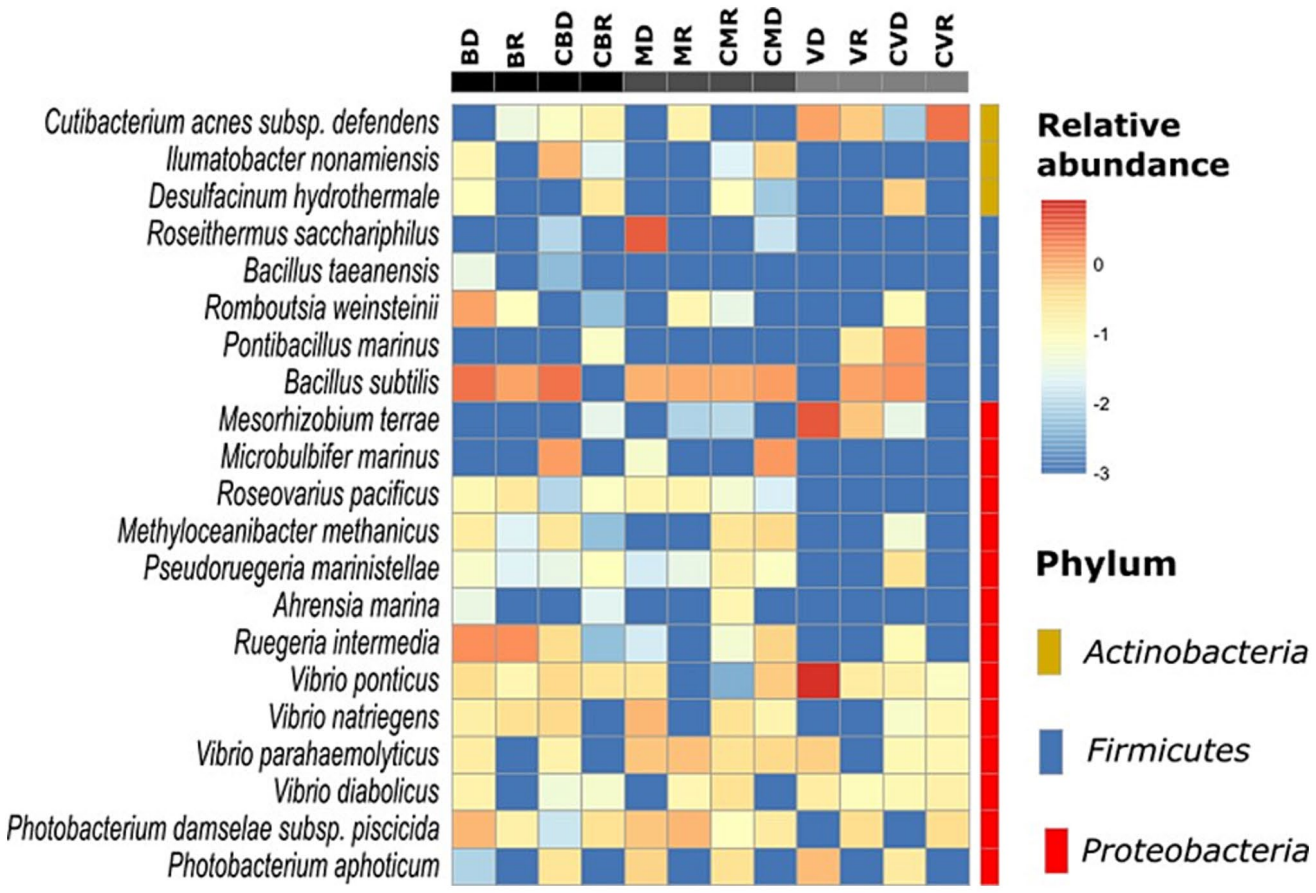
Relative abundance of major bacterial families found in sediment samples, as well as a heatmap of the most abundant species (at the ASV level), which were compared with their abundances in each group. The color intensity (log10 scale) in each panel shows the relative abundance of each species.

The effect of SHT sites on the bacterial community structure was determined using a distance matrix based on the Yue & Clayton measure and visualized using PCoA plots (Figure 4). Although the results revealed clear separation among sites, there were no differences in the community structure by season nor between the control zones with the SHT. Our analysis also revealed differences in the community structure between three SHT (Burro, Santispac, and Agua Caliente) (Figure 4). These observations were further validated using the analysis of molecular variance (AMOVA) tests, as implemented by MOTHUR, which showed that MHS sites were significantly different (*p* > 0.001). Our analysis conducted at the amplicon sequence variants (ASV) level showed the presence of bacteria typical of hydrothermal systems such as *Desulfacinum hydrothermale, Ahrensia marina, Ruegeria intermedia,* and *Vibrio diabolicus*, as well as bacteria with high relative abundance such as *Roseithermus sacchariphilus, Bacillus taeanensis, Pontibacillus marinus, and Microbulbifer marinus*. Species of the genus *Vibrio* such as *V. ponticus*, *V. natriegens*, and *V. parahaemolyticus* were also found in lower abundance (Figure 5).

## 4 Discussion

Shallow hydrothermal systems are unique ecosystems subjected to variations of different physicochemical parameters and nutrients, making them extreme environments. These conditions affect the structure of microbial communities, and various mechanisms have been developed to adapt to these conditions. In this study, the diversity of bacteria present in three areas with characteristics of hydrothermal systems was evaluated during two representative seasons, dry and rainy seasons. In addition, control sites were analyzed, which are close to shallow hydrothermal systems (SHT) but are not influenced by hydrothermal fluids or the physicochemical conditions of these extreme sites.

In the control sites, both the diversity and the bacterial richness, in general, were higher than those found in the SHT (except for the Burro site in the dry season and the Agua Caliente site in the rainy season). It was expected to obtain these results of diversity and richness because the extreme sites have specific characteristics, acting as a barrier for a large bacterial fraction that is not adapted to these physicochemical conditions.

The differences between the three hydrothermal sites studied (B, M, and V) can be explained by the characteristics of each area. At the Burro site, the temperature values that were recorded in this study were similar to those reported in a study carried out in a hydrothermal vent in the Bahía Concepción area; water and sediment temperatures were reported to be 45°C to 85°C respectively, and a pH of 6.2. It is considered that the vents in this zone have an oxidized environment because Eh values of 0.5 V were recorded, and there is evidence of the presence of Fe and Mn oxides (Dávila-Ramos et al., 2015). This hydrothermal system has been identified as a source of arsenic, a potentially toxic element (Villanueva-Estrada et al., 2013).

In contrast, the geothermal hot springs of Santispac represent a different situation, as the fluids discharge into a small lagoon surrounded by mangrove trees. Geochemical analysis of the surface marine sediments at Santispac has revealed the accumulation of priority toxic elements (PTEs) such as arsenic and mercury, particularly in the surface sediments of the mangrove lagoon, which are enriched in organic carbon (Leal-Acosta et al., 2010). High concentrations of arsenic (635 mg kg^−1^)^,^ manganese (10.3%), mercury (60.3 mg kg^−1^), barium (840 mg kg^−1^), and uranium (4.65 mg kg^−1^) were found in the crust of the hot springs near the lagoon (Leal-Acosta et al., 2010, 2018).

As Mohammad et al. (2017) comment, in these environments, organisms must cope with extreme temperatures and low nutrient availability; together, these factors reduce biodiversity, but some bacteria have developed survival strategies to adapt to such stress. Previous studies on hot springs have shown that bacterial diversity is greater when the temperature decreases (Sompong et al., 2005; Bellec et al., 2020; Barosa et al., 2023). Wang et al. (2015) observed a gradual increase in the diversity of bacteria and small eukaryotes at a greater distance from the site to the hydrothermal vent, while the diversity of archaea showed an opposite trend.

The estimated bacterial richness values ranged from 174 to 4,555, suggesting a high level of bacterial diversity compared to other large-scale nucleic acid sequencing studies in sediments. For instance, Covarrubias-Rodríguez (2016) reported richness values ranging from 506 to 894, while Wang et al. (2015) found Chao1 index values between 278 and 548 in the shallow hydrothermal vents of Kueishan Island, Taiwan. The greatest difference in terms of the richness of bacterial species concerning the SHT and control zones was found in the Santispac mangrove during the rainy season, which is also the lowest and highest values, respectively, using the Chao1 richness estimator. This hot spring waters (S) displayed high temperatures, low oxygen levels, and elevated concentrations of dissolved arsenic, mercury, and manganese. These factors are significant because they create unique microenvironments with varying conditions, particularly due to the enrichment of potentially toxic elements from the fluids mixing with seawater (Leal-Acosta et al., 2018). In the state of Baja California Sur, a very dry climate predominates (92%), with a dry and semi-dry climate (7%) and a temperate sub humid climate (1%) also occurring in a region to the south of the peninsula. The rains occur during the summer, mainly associated with the presence of hurricanes between August to November and only when they occur is there a contribution of terrestrial matter to the marine environment. Particularly in the Santispac mangrove, its location favors the removal of this type of material, depositing it mainly in front of the vent where the samples were collected.

Of the three sites sampled, the only one with information regarding studies of the non-cultivable bacterial fraction and taxonomic composition at the phyla level is that of Burro, Bahía Concepción. Dávila-Ramos et al. (2015) studied the bacterial taxonomic composition at the phyla level. These authors report the presence of the different types of *Proteobacteria* (*Gamma*, *Alpha*, *Delta*, and *Epsilon*) that represented 42% of the total that could be registered, and our result for the different types of *Proteobacteria* comprises up to 80% (*Gammaproteobacteria, Alphaproteobacteria, Deltaproteobacteria,* and *Betaproteobacteria*). Among them, *Gammaproteobacteria* and *Alphaproteobacteria* appeared as the dominant group in all stations. *Gammaproteobacteria* taxa include sulfide-oxidizing bacteria that are typically dominant in the microbial communities of both shallow- and deep-water hydrothermal vents and tend to be more prevalent in habitats with lower sulfur concentrations (Sciutteri et al., 2022). The presence of phylum Proteobacteria has been previously reported in sediments from vents of the Tyrrhenian Sea (Maugeri et al., 2010; Gugliandolo et al., 2015); Azorean Island Faial (Rajasabapathy et al., 2014); Naples, (Italy) (Bellec et al., 2020); and more recently in Levante Bay (Arcadi et al., 2023a,b; Rizzo et al., 2024). Phyla *Firmicutes*, *Chloroflexi*, and *Actinobacteria* were similar to those reported by Dávila-Ramos et al. (2015). Fagorzi et al. (2019) report the predominance of *Actinobacteria, Proteobacteria*, and *Firmicutes* phyla in Vulcano Island. In our study, *Firmicutes* and *Actinobacteria* were also detected among the main components of the sediment bacterial communities, with higher abundance, but they were not predominant. The only phylum that appeared in a lower percentage compared to the data of Dávila-Ramos et al. (2015) was *Bacteroidetes*.

The study also mentions that redox conditions influence the presence or absence of bacterial species from the classes Gammaproteobacteria, Deltaproteobacteria, and Proteobacteria, as well as from the phylum Bacteroidetes. Under the oxidizing conditions of the Bay, the phylum Bacteroidetes was found in all the analyzed samples, with concentrations ranging from 0.8 to 15.20%.

Members of the phylum *Bacteroidetes* have colonized many different ecological niches where they realized various biological functions. They are well-known as degraders of polymeric organic matter. The wide variety of habitats reflects the participation of *Bacteroidetes* in biogeochemical processes; in marine and terrestrial environments, they are involved in the decomposition of peptides and polysaccharides (Thomas et al., 2011; Fernández-Gómez et al., 2013). The above could explain why this study’s taxonomic composition at the phyla level is very similar to that reported by Dávila-Ramos et al. (2015). *Rhodothermaeota* phylum frequently was found in environments extremophilic concerning salt, temperature, or pH; they are aerobes or facultative anaerobes that require NaCl for growth (Munoz et al., 2016).

Rajasabapathy et al. (2018) analyzed the structure of the bacterial community obtained from the sediments of a hydrothermal vent in Espalamaca, Azores Island, Portugal, and compared it with other shallow-water hydrothermal vents in other regions rich in CO_2_ emissions such as South Tonga Arc and Bahía Concepción, and the comparative study revealed that *Proteobacteria* (especially *Gammaproteobacteria* and *Deltaproteobacteria*) they were predominant and that together with the *Actinobacteria* they contributed 50%.

Members of the Calditrichaceae were observed abundant in BD and BR. This species inhabits submarine vents, either shallow water or deep sea (Arcadi et al., 2023a,b). The family belongs to the class Calditrichia; these are neutrophilic, moderately thermophilic, and anaerobic bacteria that grow by fermentation of peptides or carbohydrates (Bonch-Osmolovskaya and Kublanov, 2021). Some representatives can grow by nitrate respiration using molecular hydrogen or acetate as the electron donors and forming ammonium as the end product (Kublanov et al., 2017).

Among the bacterial species found in this study, *Desulfacinum hydrothermale* stands out for its high relative abundance in the three MHS. This thermophilic, sulfate-reducing species thrives at temperatures between 37°C and 64°C and has been isolated from hydrothermal vents in Greece (Sievert and Kuever, 2000). The presence of the *Desulfacinum* genus at MHS indicates that thermophilic sulfate reduction plays a key role in the degradation of organic compounds at hydrothermal vent sites (Sievert and Kuever, 2000). Also, the presence of *Ahrensia marina* in all three systems aligns with the findings of Karuza et al. (2012), who isolated this species from shallow hydrothermal vents dominated by CO2. This genus, belonging to the order Rhodobacteriales, is typically aerobic, and photoheterotrophic, and plays a role in nitrogen and sulfur metabolism (Li et al., 2018).

Several species of the genus *Vibrio* were also detected in the studied locations, a finding consistent with reports from other researchers. For instance, Bosi et al. (2024) isolated ten *Vibrio* strains from deep-sea hydrothermal vents in the Pacific Ocean, including *V. diabolicus*. Through functional genomic analysis, the authors identified genes that may be associated with deep-sea survival and stress responses.

## 5 Conclusion

This research revealed differences in community structure among the three hydrothermal locations: Burro, Santispac, and Agua Caliente. The presence of *Gammaproteobacteria*, *Alphaproteobacteria*, *Deltaproteobacteria*, and *Betaproteobacteria* highlights their key roles in primary production within shallow hydrothermal systems, these microbial communities demonstrate their capacity to colonize diverse substrates. This study enhances the microbiological understanding of hydrothermal environments in Baja California Sur, and molecular analysis of unculturable microbes could provide further insights into their physiology and ecological roles in shallow hydrothermal systems.

## Data Availability

The datasets presented in this study can be found in online repositories. The names of the repository/repositories and accession number(s) can be found below: https://www.ncbi.nlm.nih.gov/, PRJNA1023234.

## In memoriam

In memory of Dr. José Manuel Borges-Souza, a brilliant marine biologist, passionate diver, and invaluable companion in our work endeavors. His love for the ocean and his tireless curiosity to unravel its mysteries inspired us all. Every dive and every shared discovery will remind us of his legacy, dedication, and adventurous spirit. Although the depths no longer reflect his presence, his mark will remain alive in our lives and in the waters he loved so much.
